# Secretions from the ventral eversible gland of *Spodoptera exigua* caterpillars activate defense-related genes and induce emission of volatile organic compounds in tomato, *Solanum lycopersicum*

**DOI:** 10.1186/1471-2229-14-140

**Published:** 2014-05-20

**Authors:** Simon Zebelo, Jill Piorkowski, Joseph Disi, Henry Fadamiro

**Affiliations:** 1 Department of Entomology & Plant Pathology, Auburn University, Auburn, AL 36849, USA

**Keywords:** VEG, Enzymatic activity, VOCs, Defense-related genes

## Abstract

**Background:**

Plant induced defense against herbivory are generally associated with metabolic costs that result in the allocation of photosynthates from growth and reproduction to the synthesis of defense compounds. Therefore, it is essential that plants are capable of sensing and differentiating mechanical injury from herbivore injury. Studies have shown that oral secretions (OS) from caterpillars contain elicitors of induced plant responses. However, studies that shows whether these elicitors originated from salivary glands or from other organs associated with feeding, such as the ventral eversible gland (VEG) are limited. Here, we tested the hypothesis that the secretions from the VEG gland of *Spodoptera exigua* caterpillars contain elicitors that induce plant defenses by regulating the expression of genes involved in the biosynthesis of volatile organic compounds (VOCs) and other defense-related genes. To test this hypothesis, we quantified and compared the activity of defense-related enzymes, transcript levels of defense-related genes and VOC emission in tomato plants damaged by *S. exigua* caterpillars with the VEG intact (VEGI) versus plants damaged by caterpillars with the VEG ablated (VEGA).

**Results:**

The quantified defense-related enzymes (i.e. peroxidase, polyphenol oxidase, and lipoxigenase) were expressed in significantly higher amounts in plants damaged by VEGI caterpillars than in plants damaged by VEGA caterpillars. Similarly, the genes that encode for the key enzymes involved in the biosynthesis of jasmonic acid and terpene synthase genes that regulate production of terpene VOCs, were up-regulated in plants damaged by VEGI caterpillars. Moreover, the OS of VEGA caterpillars were less active in inducing the expression of defense genes in tomato plants. Increased emissions of VOCs were detected in the headspace of plants damaged by VEGI caterpillars compared to plants damaged by VEGA caterpillars.

**Conclusion:**

These results suggest that the VEG of *S. exigua* caterpillars contains elicitors of late plant defense signaling in tomato which trigger defense-related enzymatic activity, regulate expression of defense-related genes, and induce emission of plant VOCs. These signaling cascades may have important ramifications for plant-insect and tritrophic interactions.

## Background

Plants have evolved to defend themselves against biotic stressors such as insects and pathogens. Various insect secretions including oviposition fluids, oral secretions (OS), and insect excreta are known to act as elicitors of induced plant defenses against insect herbivory [1-5]. Plant defense signaling cascades induced by insects begin with plant recognition of insect-derived elicitors followed by plasma trans-membrane potential (V_m_) depolarization [6-8], the rise in cytosolic calcium ions [9] and a burst of reactive oxygen species (ROS), including hydrogen peroxide (H_2_O_2_) and nitric oxide (NO) [2,10,11]. These cascades lead to a rise in production of the phytohormone, jasmonic acid (JA) and salicylic acid (SA) [3,12] that regulate the transcript level of defense-related genes [3,13], and end with metabolic changes including release of volatile organic compounds (VOCs) [1,3,13-15] and production of toxic compounds in the plants [16,17]. Plasma trans-membrane potential (V_m_) depolarization, rise in cytosolic calcium ions and a burst of reactive oxygen species (ROS) which occurs from seconds to hour/s after insect damage referred to as early plant defense responses, while production of the phytohormone, change in transcript level of defense-related genes and metabolic changes including release of VOCs and production of toxic compounds which occurs from hour/s to day/s after insect damage referred to as late plant defense responses [18].

Foliar feeding insects ingest leaves by snipping plant material continuously. This process causes a series of mechanical injury, usually supplemented with introduction of oral secretions into the damaged tissue [1,3,4,10,18,19]. It is vital for plants to differentiate mechanical injury from herbivore damage and change these different biotic stress signals into suitable physiological responses. Studies have shown that plants are able to differentiate simple mechanical injury from herbivore injury [6,10,20-25]. Investigations at the molecular level have revealed different gene expression patterns of defense-related genes in plants with mechanical injury versus plants damaged by insects [6,20-25]. Application of insect OS to mechanical injury can mimic most plant responses to herbivory [6,22,26], showing that the OS constitute elicitors by which plants recognize insect attack [3,6,26,27]. Indeed, several elicitors have been isolated from insect OS that trigger plant defenses against herbivory, such as β-glucosidase [15], volicitin, a fatty acid–amino acid conjugate [1,28,29], caeliferins [30], and inceptins [25]. Lepidopteran OS consists of saliva from mandibular and labial secretions, and regurgitant from digestive tract [19,31]. The OS deposited on herbivore fed plant material also contains secretions from the ventral eversible gland (VEG) [32]. Despite the discovery of several elicitors, studies that show whether these elicitors originated from salivary glands or from other organs associated with feeding, such as the ventral eversible gland (VEG) are limited. Volicitin originated from the gut tissues of *Spodoptera litura* larvae [33] and inceptins are partially digested chloroplast protein formed when *Spodoptera frugiperda* attack cowpea [25].

The VEG is a secretory structure found on the ventral surface of the thorax of caterpillars (lepidopteran larvae). It consists of two regions with different functions: a non-eversible glandular sac lined with secretory cells and an eversible cuticular tube. Eversion of the cuticular tube forms a visible papilla, whereas secretions from the secondary gland area on the cuticular tube are transferred to the apex of the papilla and released [34]. Since the tip of the everted VEG can reach the mandibles [35], its secretions are deposited onto the food substrate with the OS [32]. Secretions from the VEG of caterpillars have been associated with defense against predators and the production of anti-aggregation pheromones [34-36]. However, the role of VEG secretions in plant-insect interactions remains unclear. Recently, Zebelo and Maffei [32] demonstrated that secretions from the VEG of *Spodoptera littoralis* caterpillars trigger early defense signaling events in *Arabidopsis thaliana.*

In the present study, we investigated possible involvement of VEG secretions from *S. exigua* caterpillars in the induction of late defense signaling in tomato. We quantified and compared the activity of defense-related enzymes, transcript levels of terpene synthase genes and other defense-related genes, and VOC emission in tomato plants damaged by *S. exigua* caterpillars with the VEG intact (VEGI) versus plants damaged by caterpillars with the VEG ablated (VEGA) as well as mechanically injured plants treated with OS from VEGI caterpillars (MI + OSVEGI) versus mechanically injured plants treated with OS from VEGA caterpillars (MI + OSVEGA).

## Results

### VEG ablation didn’t affect *S. exigua* feeding activity

Before we started to assess the impact of VEG secretions on triggering plant defense, we evaluated whether VEG ablation affects feeding activity of *S. exigua* caterpillars. There were no significant differences between VEGA (2.42 ± 0.44 cm^2^) and VEGI (2.61 ± 1.04 cm^2^) caterpillars on leaf area consumption after 24 h (P > 0.84).

### VEG secretions activate defense-related enzymes in tomato

**Figure 1:**
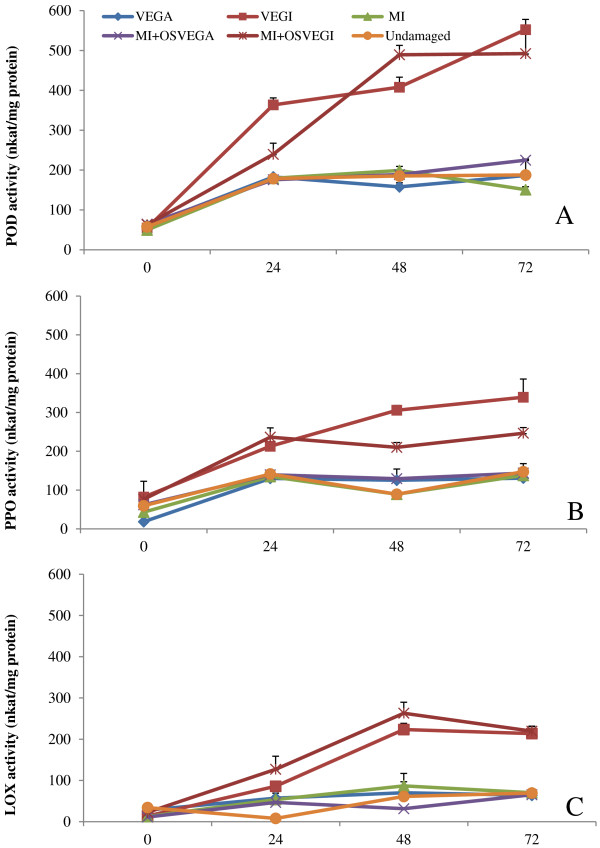
**Secretions from the ventral eversible gland (VEG) of *Spodoptera exigua *caterpillars activate defense-related enzymes in tomato.** Figure shows activity (expressed as mean ± SEM nkat/mg protein) of three defense-related enzymes, **(A)** peroxidase (POD), **(B)** polyphenol oxidase (PPO), and **(C)** lipoxygenase (LOX), in leaves of tomato plants damaged by caterpillars with the VEG intact (VEGI), plants damaged by caterpillars with the VEG ablated (VEGA), mechanically injured (MI) plants, mechanically injured plants treated with oral secretion (OS) from VEGI caterpillars (MI + OSVEGI), mechanically injured plants treated with OS from VEGA caterpillars (MI + OSVEGA), and undamaged (control) plants, at 0, 24, 48 and 72 h after caterpillar feeding. Data were collected from three plants (i.e. 3 biological replicates) per treatment (see Table 1 for significant differences among treatments).

The selected defense-related enzymes, peroxidase (POD), polyphenol oxidase (PPO), and lipoxygenase (LOX), were expressed in significantly higher amounts in plants damaged by VEGI caterpillars and MI + OSVEGI than in plants damaged by VEGA caterpillars, mechanically injured (MI) plants, MI + OSVEGA plants, and untreated (control) plants. Activity of POD was significantly higher in VEGI-damaged and MI + OSVEGI tomato plants than in VEGA-damaged, MI, MI + OSVEGA or undamaged plants, starting as early as 24 h after treatment (Figure 1A). Activity of PPO 48 h after treatment was 8.2, 9.1, 8.8 and 8.5% higher in plants damaged by VEGI caterpillars than in plants damaged by VEGA caterpillars, MI, MI + OSVEGA, or undamaged plants, respectively (Figure 1B). A significant increase in LOX-specific activity levels was detected as early as 24 h after treatment in plants damaged by VEGI caterpillars and MD + OSVEGI plants compared to the other treatments. Activity of LOX 72 h after treatment was 14.2, 17, 14.6 and 21.6%, higher in plants damaged by VEGI caterpillars than in plants damaged by VEGA caterpillars, MI, MI + OSVEGA, or undamaged plants, respectively (Figure 1C). In general, no significant differences were recorded in enzymatic activity between plants damaged by VEGI caterpillars and MI + OSVEGI plants (Figure 1 and Table 1).

**Table 1:** Levels of defense-related enzymes in tomato plants in response to six treatments

**Hours after treatment**	**Treatment**	**Enzymatic activity (nkat/mg protein)**		
		**POD**	**PPO**	**LOX**
**0**	VEGI	55.29 ± 2.95a	52.35 ± 4.65a	13.54 ± 0.66a
	VEGA	61.17 ± 1.35a	58.23 ± 0.89a	28. 45 ± 6.54a
	MI + OSVEGI	66.46 ± 1.14a	56.42 ± 2.80a	22.71 ± 1.12a
	MI + OSVEGA	64.60 ± 4.38a	62.39 ± 11.44a	11.20 ± 0.88a
	MI	49.70 ± 0.75a	59.25 ± 5.49a	12.97 ± 3.48a
	Undamaged	56.48 ± 1.14a	60.51 ± 3.47a	34.80 ± 2.14a
**24**	VEGI	363.60 ± 17.66a	212.08 ± 20.20b	85.84 ± 12.59a
	VEGA	182.81 ± 3.97c	130.06 ± 8.76c	57.67 ± 3.22b
	MI + OSVEGI	239.45 ± 24.23b	236.78 ± 23.97a	127.54 ± 6.61a
	MI + OSVEGA	175.78 ± 5.79c	139.45 ± 4.65c	46.56 ± 1.71b
	MI	179.29 ± 21.89c	134.82 ± 25.09c	53.33 ± 5.24b
	Undamaged	178.70 ± 1.22c	141.43 ± 6.76c	22.05 ± 0.61b
**48**	VEGI	407.96 ± 17.10b	305.29 ± 17.47a	70.75 ± 2.06a
	VEGA	157.79 ± 10.50d	125.63 ± 22.31c	33.95 ± 2.89d
	MI + OSVEGI	489.27 ± 23.70a	210.60 ± 7.35b	57.06 ± 2.70b
	MI + OSVEGA	188.80 ± 11.67c	129.27 ± 6.05c	17.17 ± 2.22c
	MI	199.16 ± 10.30c	89.65 ± 68.88c	31.18 ± 5.37d
	Undamaged	195.25 ± 7.41c	89.63 ± 17.64c	27.47 ± 6.49d
**72**	VEGI	552.26 ± 24.75a	339.99 ± 21.92a	213.67 ± 35.32a
	VEGA	186.40 ± 7.67e	130.76 ± 24.01c	63.78 ± 8.84b
	MI + OSVEGI	492.27 ± 17.01b	246.12 ± 15.96b	219.46 ± 9.81a
	MI + OSVEGA	224.92 ± 12.97c	143.74 ± 8.34c	65.38 ± 3.07b
	MI	150.80 ± 8.22d	137.80 ± 5.03c	70.18 ± 1.85b
	Undamaged	187.66 ± 18.53e	147.32 ± 20.83c	69.45.68 ± 1.09b

Table shows activity (expressed as mean ± SEM nkat/mg protein) of three defense-related enzymes, peroxidase (POD), polyphenol oxidase (PPO), and lipoxygenase (LOX), in tomato plants damaged by caterpillars with the VEG intact (VEGI), plants damaged by caterpillars with the VEG ablated (VEGA), mechanically injured (MI) plants, mechanically injured plants treated with oral secretion (OS) from VEGI caterpillars (MI + OSVEGI), mechanically injured plants treated with OS from VEGA caterpillars (MI + OSVEGA), and undamaged (control) plants, at 0, 24, 48 and 72 h after caterpillar feeding. Data were collected from three plants (i.e. 3 biological replicates) per treatment. Means (±SEM) within the same column and time period having different letters are significantly different (P < 0.05).

### VEG secretions induce VOCs emission in tomato

**Figure 2:**
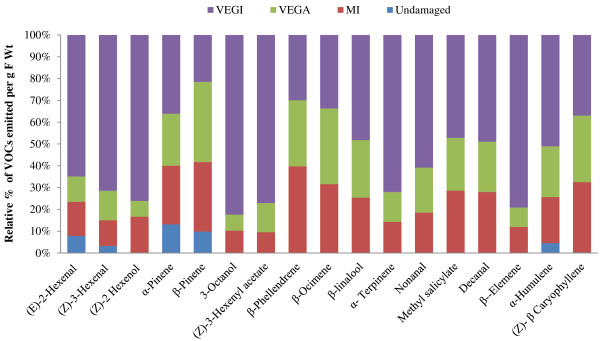
**Secretions from the ventral eversible gland (VEG) of *Spodoptera exigua *caterpillars activate emission of volatile organic compounds (VOCs) in tomato.** Figure shows emission of VOCs (expressed as % μg g^-1^ fwt) by tomato plants damaged by caterpillars with the VEG intact (VEGI), plants damaged by caterpillars with the VEG ablated (VEGA), mechanically injured (MI) plants, and undamaged (control) plants. Data were collected from three plants (i.e. 3 biological replicates) per treatment (see Table 2 for significant differences among treatments).

Key differences were recorded in the headspace VOC profiles of tomato plants from the different treatments (Figure 2). Increased emission of VOCs was detected in the headspace of plants damaged by VEGI caterpillars compared to plants damaged by VEGA caterpillars, mechanically injured (MI) plants, and untreated (control) plants. Specifically, green leaf volatiles (GLVs) and certain monoterpenes were emitted in higher amounts by plants damaged by VEGI caterpillars than in the other treatments (Figure 2, Table 2). In particular, the GLVs, (*E*)-2-hexenal, (*Z*)-3-hexenal, (*Z*)-3-hexenyl acetate and (*Z*)-2-hexenol were emitted in 7-fold, 5-fold, 7-fold and 10-fold, respectively, in plants damaged by VEGI caterpillars compared to plants damaged by VEGA caterpillars (Figure 2 and Table 2).

**Table 2:** Quantitative analysis of emission of volatile organic compounds (VOCs)

**VOCs**	**KI**	**VEGI**	**VEGA**	**MI**	**Undamaged**
(*E*)-2-Hexenal	855	14.10(1.94)a	2.53(0.99)b	3.40(0.93)b	1.7(0.32)b
(*Z*)-3-Hexenal	865	10.27(0.19)a	1.94(0.33)b	1.70(0.24)b	0.46(0.03)b
(*Z*)-2 Hexenol	858	13.07(2.53)a	1.24(0.03)b	2.86(0.20)b	nd
α-Pinene	939	15.867(1.40)a	10.47(0.78)a	11.82(1.09)a	5.76(0.01)b
β-Pinene	979	1.34(0.54)a	2.29(0.64)a	1.99(0.14)a	0.61(0.20)b
3-Octanol	991	27.39(1.27)a	2.43(0.24)b	3.42(0.60)b	nd
(*Z*)-3-Hexenyl acetate	1002	10.09(1.77)a	1.75(0.16)b	1.25(1.34)b	nd
β-Phellendrene	1030	19.79(1.91)a	20.01(1.57)a	26.27(1.85)a	nd
β-Ocimene	1040	16.58(1.23)a	17.07(1.31)a	15.52(0.36)a	nd
β-linalool	1026	19.52(1.70)a	10.66(0.77)b	10.30(1.66)b	nd
α- Terpinene	1060	16.79(1.03)a	3.19(0.72)b	3.33(1.17)b	nd
Nonanal	1101	10.82(1.65)a	3.66(1.74)b	3.30(0.51)b	nd
Methyl salicylate	1190	13.14(1.06)a	6.73(0.32)b	7.97(0.47)b	nd
Decanal	1202	174.38(1.63)a	82.27(2.54)b	99.68(4.64)b	nd
δ--Elemene	1338	32.85(1.20)a	3.69(0.03)b	4.96(0.71)b	nd
β- Elemene	1319	19.89(5.46)a	9.06(1.22)b	8.24(0.52)b	1.76(0.21)c
(*Z*)- β- Caryophyllene	1455	106.94(3.61)a	88.33(10.91)b	93.10(3.13)b	0.86(0.06)c

Table shows emission of VOCs ( μg g^-1^ fwt) by tomato plants damaged by caterpillars with the VEG intact (VEGI), plants damaged by caterpillars with the VEG ablated (VEGA), mechanically injured (MI) plants, and undamaged (control) plants.Data were collected from three plants (i.e. 3 biological replicates) per treatment. Means (±SEM) within the same row having different letters are significantly different (*P* < 0.05). Kovats retention index (KI) is indicated for each compound. *nd* = not detected.

The monoterpenes, β-linalool and γ-terpinene, were emitted in significantly higher amounts by plants damaged by VEGI caterpillars compared to plants damaged by VEGA caterpillars and the other treatments (Figure 2, Table 2). However, no significant differences were recorded among the treatments in the emission of α-pinene, β-phellendrene, β-pinene and β-ocimene. The sesquiterpenes, (*E*)-β-caryophyllene, α-humulene and β-elemene, were also emitted in significantly higher amounts by plants damaged by VEGI caterpillars compared to the other treatments. Furthermore, emission of the fatty acids (3-octanol, nonanal) and organic ester (methyl salicylate), was higher in VEGI-damaged plants (Figure 2 and Table 2).

### VEG secretions increase transcript levels of defense-related genes in tomato

We used quantitative RT-PCR to quantify the transcript levels of six defense-related genes including genes encoding lipoxygenase (*LOX*), allene oxide synthase (*AOS*), and four genes involved in terpene biosynthesis (terpene synthase genes). *LOX* and *AOS* are key enzymes in the jasmonic acid (JA) biosynthesis pathway. Most of defense-related genes were found up-regulated in plants damaged by VEGI caterpillars and in MI + OSVEGI plants compared to plants damaged by VEGA caterpillars, mechanically injured (MI), MI + OSVEGA, or untreated (control) plants (Table 3). In particular, the transcript levels of the terpene synthase genes, *TPS5* (encodes monoterpene synthesis) [37], and *TPS12* (catalyzes formation of the sesquiterpenes (*E*)-β-caryophyllene and α-humulene) [38], were significantly higher in plants damaged by VEGI caterpillars and MI + OSVEGI plants compared to the other treatments.

**Table 3:** Gene expression results

	**VEGI**	**VEGA**	**MD + OSVEGI**	**MD + OSVEGA**	**MD**	**Control**
*LOX*	4.47(0.46)b	0.14(0.15)b	4.65(1.30)a	1.13(0.08)b	0.95(0.12)b	1.04(0.05)b
*AOS*	6.01(0.18)b	1.12(0.21)c	6.85(1.22)a	0.96(0.07)c	0.96(0.16)c	1.01(0.01)c
*TPS5*	4.68(0.39)a	0.54(0.07)b	5.31(0.81)a	1.13(0.17)c	0.75(0.16)c	1.08(0.06)c
*TPS7*	2.32(0.06)a	2.19(0.19)a	1.58(0.91)a	2.11(0.08)a	2.02(0.05)a	1.13(0.16)b
*TPS12*	5.57(0.74)a	0.86(0.23)b	6.55(0.85)a	0.90(0.27)b	0.91(0.26)b	1.02(0.13)b
*TPS25*	2.03(0.28)a	2.43(0.09)a	2.21(0.43)a	2.03(0.10)a	2.14(0.06)a	1.13(0.16)b

Differential expression of genes involved in jasmonic acid (JA) and terpene biosynthesis in tomato plants damaged by caterpillars with the VEG intact (VEGI), plants damaged by caterpillars with the VEG ablated (VEGA), mechanically injured (MI) plants, mechanically injured plants treated with oral secretion (OS) from VEGI caterpillars (MI + OSVEGI), mechanically injured plants treated with OS from VEGA caterpillars (MI + OSVEGA), and undamaged (control) plants. qRT-PCR analyses are shown as fold change in expression. Means (±SEM) within the same row having different letters are significantly different (*P* < 0.05).

## Discussion

Tomato plants damaged by *S. exigua* caterpillars with intact ventral eversible gland (VEGI) expressed significantly higher amounts of defense-related enzymes and genes, and headspace VOCs than plants damaged by caterpillars with ablated VEG (VEGA). These results suggest that secretions from the VEG of *S. exigua* caterpillars contain elicitors of late defense signaling in tomato. To our knowledge, this is the first report on the role of caterpillar VEG secretions as an elicitor of late defense signaling in plants. A previous study by Zebelo and Maffei [32] showed that VEG secretions of *Spodoptera littoralis* caterpillars induced early defense signaling in *Arabidopsis thaliana*.

The three defense-related enzymes, peroxidase (POD), polyphenol oxidase (PPO), and lipoxigenase (LOX), were expressed in significantly higher amounts in plants damaged by VEGI caterpillars or mechanically injured plants treated with oral secretion (OS) from VEGI caterpillars (MI + OSVEGI) than in plants damaged by VEGA caterpillars, mechanically injured (MI) plants, mechanically injured plants treated with oral secretion (OS) from VEGA caterpillars (MI + OSVEGA), or untreated (control) plants. All three enzymes are components of the octadecanoid signal transduction pathway, which regulates the production of the phytohormone, jasmonic acid (JA) [39-41]. Peroxidases (PODs) are a group of plant defense-related enzymes, which limit plant nutritional quality to insect herbivores through quinone and reactive oxygen species generation with subsequent inhibition of insect digestion of plant material [41,42]. Over-expression of PODs can enhance plant resistance to insects [43] and limit plant nutritional quality to insect herbivores [41]. Suzuki *et al*. [41] reported that herbivory by caterpillars and high POD activity resulted in the up-regulation of several tomato genes including genes encoding proteinase inhibitors.

Polyphenol oxidase (PPO) is an inducible enzyme that is found throughout the plant kingdom and known to have defensive role against herbivores [40,44] and pathogens [45,46]. Production of PPO is induced by mechanical injury, methyl jasmonate (MeJa) and herbivory [39]. Similar to our results, Chung *et al*. [47] reported high PPO levels in tomato plants wounded mechanically and treated with oral secretions (OS) from Colorado potato beetle, *Leptinotarsa decemlineata*, suggesting that insect OS contain elicitors of PPO activity [47]. Lipoxygenases (LOXs) are another group of anti-oxidative enzymes involved in plant defense against herbivory and pathogens through the octadecanoid pathway [48]. One of the most important functions of LOX in plant defense is the oxidation of linolenic acid in the JA signaling pathway [49]. Allene oxide synthase (*AOS*) catalyzes the first step of the LOX pathway that leads to JA biosynthesis [49]. In the present study, we observed an early induction of LOX-specific activity within 24 h of feeding by caterpillars with intact VEG (VEGI). Likewise, the transcript levels of *LOX* and *AOS* genes were higher in plants damaged by VEGI caterpillars compared to plants damaged by caterpillars with ablated VEG (VEGA). These results are consistent with previous studies which demonstrate that caterpillar feeding up-regulates the expression of *LOX* genes in tomato [50].

Our results also showed increased emission of VOCs in tomato plants damaged by VEGI caterpillars compared to plants damaged by VEGA caterpillars or mechanically injured plants. Among the common VOCs induced by herbivory are those that are LOX-derived, such as green leaf volatiles (GLVs), terpenoids and methyl salicylate [51]. Numerous plants emit GLVs and other VOCs as an indirect defense strategy against herbivory, as these volatiles can attract predacious and parasitic natural enemies of herbivores [52-54]. In this study, GLVs and certain monoterpenes were emitted in higher amounts by plants damaged by VEGI caterpillars, suggesting the involvement of the VEG in the induction of plant VOCs. For instance, most GLVs including (*E*)-2-hexenal, (*Z*)-3-hexenal, (*Z*)-2-hexenol and (*Z*)-3-hexenyl acetate were detected in higher amounts in plants damaged by VEGI caterpillars compared to plants damaged by VEGA caterpillars. Interestingly, many of these GLVs are used as host location cues by caterpillar parasitoids [55], suggesting that VEG secretions may impact tritrophic interactions. GLVs have also been reported to play a role in plant-plant interactions [56].

Another highly diverse group of plant compounds are the terpenoids, which are synthesized by a group of enzymes called terpene synthases (*TPS*) to produce mono-, sesqui- and diterpenes [51]. Terpenes are more costly to synthesize per gram than most other primary and secondary plant compounds [57]. Studies have shown that a single mechanical plant tissue injury event may not elicit induced defense related volatile organic compounds (VOCs) [58,59]. However, application of OS to mechanically wounded site could elicit the release of inducible volatile compounds and thereby mimic herbivory [58,59]. In the present study, we observed significantly higher emission of the monoterpenes, β-linalool and γ-terpinene, in plants damaged by VEGI caterpillars compared to those damaged by VEGA caterpillars. However, there were no significant differences recorded among the treatments in the emission of other monoterpenes such as α-pinene, β-phellendrene, β-pinene and β-ocimene, suggesting that not all VOCs are inducible by VEG secretions.

Like monoterpenes, sesquiterpenes are phytoalexins which play a pivotal role in direct and indirect defenses against herbivores [60]. In the present study, several sesquiterpenes (i.e. (*E*)-β-caryophyllene, α-humulene and β-elemene), some fatty acids (3- octanol and nonanal) and an organic ester (methyl salicylate) were emitted in significantly higher amounts by plants damaged by VEGI caterpillars compared to plants damaged by VEGA caterpillars.

Results from gene expression studies showed that most terpene synthase genes (i.e. *TPS7* which encodes the monoterpene, β-Ocimene and *TPS12* which encodes the sesquiterpenes, (*E*)-β-caryophyllene and α-humulene) and the genes involved in the biosynthesis of GLVs and jasmonic acid (i.e. *LOX* and *AOS*) were up-regulated in plants damaged by VEGI caterpillars as well as in mechanically injured plants treated with oral secretion from VEGI caterpillars (MI + OSVEGI). However, the transcript levels of these genes were not up-regulated in plants damaged by VEGA caterpillars, mechanically injured plants treated with oral secretion from VEGA caterpillars (MI + OSVEGA), or mechanically injured (MI) plants. These results suggest that an intact VEG in *S. exigua* caterpillars is crucial for eliciting late defense signaling via the expression of defense-related genes. These findings are in agreement with those of Bricchi *et al.*[3] which showed that mechanical injury alone failed to increase the transcript levels of terpene synthase and JA biosynthesis genes in *Arabidobsis thaliana,* but mechanical injury treated with *Spodoptera littoralis* oral secretion activated the genes.

In a recent review of the role of caterpillar secretions on induced plant defenses, Felton [61] suggested that the VEG may play an important role in secretion during feeding by caterpillars in the family Noctuidae. The structure and proximity of the VEG to the caterpillar mouthparts lend credence to this proposal. When a caterpillar feeds on a plant material the VEG is distended from its eversible position on the ventral surface of the caterpillar thorax and reaches the injured plant surface [32,61]. Furthermore, because the tip of the everted VEG can reach the mandibles during feeding [35], the VEG secretions are usually deposited onto the food substrate with the OS [32]. Our results confirm that the VEG secretions, which are deposited along with oral secretions or regurgitate onto plants during caterpillar feeding can induce late defense signaling in tomato. Further studies are needed to identify the bioactive components of the VEG secretions that trigger plant defense signaling.

## Conclusion

The VEG was first reported in 1745 [62], but very little is known about its role in plant-insect interactions. Our current results suggest that the VEG of *S. exigua* contain elicitors of late plant defense signaling which may trigger defense-related enzymatic activity, regulate expression of terpene synthase genes and other defense-related genes, and induce plant VOCs, with potential ramifications for plant-insect and tritrophic interactions. Studies are underway in our lab to investigate whether the VEG secretion alone or in combination with other labial gland secretions and gut regurgitates trigger plant responses against insect herbivory. Further studies are needed to comprehend the complexity of plant signaling networks and the role of insect oral secretions in mediating plant-insect and trititrophic interactions.

## Methods

### Plant and animal material

Tomato plants (*Solanum lycopersicon* Mill. cv Microtom) were grown from seeds in plastic pots with sterilized sunshine mix soil at 23°C and 60% relative humidity using daylight fluorescent tubes (270 μmol m^-2^ s^-1^) with a light phase of 16 h. Six weeks old non-flowering potted tomato plants were used for the experiments. *Spodoptera exigua* eggs purchased from Benzon Research (Carlisle, PA) were used to start laboratory colonies at Auburn University (Auburn, AL). Caterpillars were fed a laboratory-prepared pinto bean diet and maintained at 25 ± 1°C, 75 ± 5% relative humidity, and 14:10-h (L/D) photoperiod.

### VEG ablation and oral secretion collection

**Figure 3:**
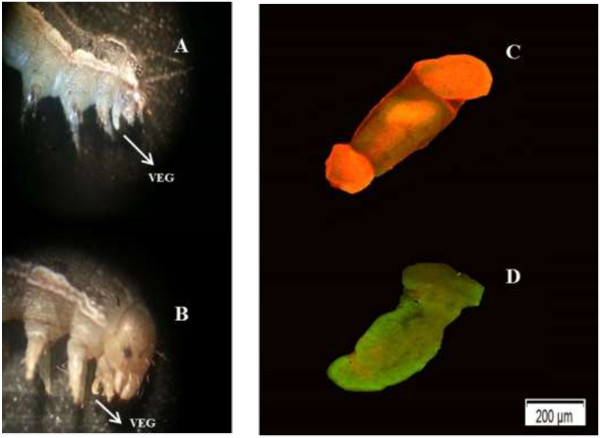
**Micrographs of the ventral eversible gland (VEG) of *Spodoptera exigua *caterpillar.** VEG treated with heat **(A)**, Intact VEG – not treated with heat **(B)**, Dead VEG due to heat treatment (ethidium homodimer-1 is well retained in dead VEG cells, producing a bright red fluorescence **(C)**, and Intact VEG polyanionic dye calcein is well retained in live VEG cells, producing an intense uniform green fluorescence **(D)**. Magnification = 250X.

VEG ablation (VEGA) was done as previously described in Zebelo and Maffei [32] with little modification. Third-instar larvae were chilled on ice until they became inactive. Using stainless steel pins each caterpillar was held in a styrofoam comb by bending the pins against its body. The styrofoam with caterpillar was placed under olympus stereomicroscope (Tokyo, Japan) set at magnification of 250x. The caterpillar head was gently pushed backwards with cotton ear buds to evert the VEG, and a stainless steel pin was heated with a Bunsen flame until it turns glowing red and then brought close to the everted VEG. The VEG was turned to a whitish-milky color after heat treatment (Figure 3A), and after ablation VEGs were not regenerated after molting. VEGA larvae were allowed to feed for 24 h on tomato leaves. Control larvae (i.e. larvae with VEG intact, VEGI) were chilled and placed in a styrofoam comb, elicited to evert the VEG, but not treated with the heated pin (Figure 3B). VEGI larvae were also allowed to feed for 24 h on tomato leaves. Third-instar VEGA and VEGI caterpillars were allowed to molt to the fourth and fifth instars. This allowed them to acclimate to the host plant, recover from the ablation and resume feeding prior to the tests. To compare the level of damage caused by VEGA and VEGI caterpillars, Leaves were excised from tomato plants and placed in 8 cm diameter Petridish carpeted with moist white paper towel. The excised leaves were plugged at the petiole with wet cotton balls to prevent desiccation. VEGA and VEGI caterpillars were allowed to feed on excised leaves (one larva per leaf). The portion of the leaf fed upon by the larva was quantified after one day by scanning the leaf. The scanned images were imported into Image J software (ImageJ; http://rsbweb.nih.gov/ij/) to measure the amount of leaf consumed.

Oral secretion was collected from VEGI and VEGA caterpillars as previously described in Zebelo and Maffei [32]. The OS was diluted in 5 mm 2-(*N*-morpholino) ethanesulfonic acid (Mes-NaOH) (pH 6.0) buffer at the rate 1:5 and 5 μl was applied at the site of mechanical injury (MI) in tomato leaves with a micro-syringe. The ratio of oral secretion to Mes-NaOH buffer and the amount of OS solution added to mechanical injured (MI) tomato plants were as reported in previous studies [3,6,10,11,32]. Moreover, previous studies have shown that Mes-NaOH buffer alone failed to trigger plant defense signaling [3,6,10,11,32].

### Live/Dead VEG assay

After acclimation and recovery from ablation, representative VEGI and VEGA caterpillars from the above treatments were chilled again on ice until flaccid and placed in a styrofoam comb. Using stainless steel pins each caterpillar was held in a styrofoam comb by bending the pins against its body. The caterpillar head was pushed backwards to evert the VEG and a fine-point forcep was used to remove the VEG, free of oral secretions or excess hemolymph and directly placed in microscopic concave well slides (Microscope world, Carlsbad CA, USA). The LIVE/DEAD viability/cytotoxicity assay kit (Biotium Hatward, CA, USA) was used to confirm VEG ablation. Two-color fluorescence cell viability assay was done with an Olympus fluorescence microscope (BX61, Tokyo, Japan) set at magnification 40X.

Ethidium homodimer-1 (EthD-III, which is a component of the assay kit) enters cells with damaged membranes and undergoes a 40-fold enhancement of fluorescence upon binding to nucleic acids, thereby producing a bright red fluorescence in dead cells (ex/em ~495 nm-635 nm) (Figure 3C). Live cells were distinguished by the presence of ubiquitous intracellular esterase activity, determined by the enzymatic conversion of the virtually non-fluorescent cell-permeating calcein acetoxymethyl (AM) to the intensely-fluorescent calcein. The polyanionic dye calcein is well retained within live cells, producing an intense uniform green fluorescence in live cells (ex/em ~495 nm/~515 nm) (Figure 3D). The VEG tissue labeling was done according to the manufacturer’s recommendations.

### Enzyme assays

We quantified the activity of three enzymes involved in plant defense in response to insect herbivores: peroxidase (POD), polyphenol oxidase (PPO) and lipoxygenase (LOX). Leaf samples were collected from tomato plants damaged by VEGI caterpillars, VEGA caterpillars, mechanically injured (MI) plants, mechanically injured plants treated with OS from VEGI caterpillars (MI + OSVEGI), mechanically injured plants treated with OS from VEGA caterpillars (MI + OSVEGA), and undamaged (control) plants, at 0, 24, 48 and 72 h after caterpillar feeding. Ten leaves per plant were grounded in liquid nitrogen and 0.2 g of grounded leaves from each sample was homogenized in 2 ml ice-cold 0.05 M phosphate buffer (pH 7.2 for POD, pH 7.8 for PPO) containing 1% (w/v) polyvinylpyrrolidone (PVP). The homogenate was centrifuged at 12,000 *g* for 45 min at 4°C. The supernatant was collected and used for POD and PPO assays. POD activity was determined as described in [63]. PPO activity was assayed with 0.05 M catechol as a substrate by a spectrophotometric procedure [64].

LOX activity was measured as conjugated diene formation [65]. Leaves were ground in liquid nitrogen and 0.2 g of grounded leaves from each sample was homogenized with 1 ml ice-cold 0.5 M Tris–HCl buffer (pH 7.6) and centrifuged at 12,000 *g* for 45 min at 4°C. The supernatant was kept at 4°C until used. The substrate contained 1.6 mM linoleic acid and 0.5% (v/v) Tween 20 in 0.1 M phosphate buffer (pH 7.6). The reaction was initiated by the addition of 0.2 ml of the supernatant in 4.8 ml of the substrate. Diene formation was measured as increase of absorbance at 234 nm.

Enzymatic activity was calculated by employing the linear regression equation of respective substrate production over time, on the basis of an extinction coefficient estimated with an authentic standard. The catalytic activity of the enzyme was calculated in katal (Kat), which is defined as the amount of enzyme that catalyzes the formation of 1 mol of substrate s^-1^ under the above assay conditions. Protein concentration was quantified by the method of Bradford [66] using bovine serum albumin as the standard. The data were analyzed using one-way ANOVA followed by the Tukey-Kramer HSD multiple comparison test at a significance level of *P* < 0.05.

### Collection of VOCs from tomato plants damaged by VEGI versus VEGA caterpillars

To determine the role of the VEG on VOC emission in tomato, headspace volatiles were collected from plants damaged by VEGI caterpillars, VEGA caterpillars, mechanically injured (MI) plants, and undamaged (control) plants. Fifteen 3^rd^ instar *S. exigua* caterpillars (VEGI or VEGA) were allowed to feed on a potted tomato plant for 24 h. Feeding by these caterpillars for 6 h resulted in ~ 25-35% leaf area damage, which is similar to the mechanical injury simulation with pattern wheel, as previously described in [13]. The pot with the potting soil was wrapped with aluminum foil to minimize evaporation of water and volatiles from the soil and placed in a volatile collection chamber consisting of a 5 L glass jar. A purified (using activated charcoal) air stream of 350 ml/min was passed through the jar at room temperature for 24 h and plants were illuminated with fluorescent light bulbs generating 50 μmol m^-2^ s^-1^ with a photoperiod of 16 h. Headspace volatiles were collected using a trap containing 50 mg of Super-Q (Alltech Associates, Deerfield, IL) and eluted with 300 μl of methylene chloride. The resulting extracts (300 μl) were stored in a freezer (at -20°C) until use. Another container with potting soil without plant or caterpillars was used to check for miscellaneous impurities and breakthrough of the trap during sampling. One microliter of each headspace volatile extract was analyzed by gas-chromatography (Agilent Technologies, mod. 7890A) coupled with mass spectrometry (Agilent technologies, mod. 5975C), as described in [13]. Compounds were identified by comparison of their mass spectra and retention indices (Kováts index) with those of reference substances and by comparison with the NIST mass spectral search software v 2.0 using the NIST 05 library (National Institute of Standards and Technology, Gaithersburg, MD, USA). External calibration curves were made with standard solutions of (*E*)-2-hexenal, α-pinene and (*E*)-β-caryophyllene for quantitative measurements, as previously described in [13]. The data were analyzed by using one-way ANOVA followed by the Tukey-Kramer HSD multiple comparison test at a significance level of *P* < 0.05.

### Total RNA isolation and cDNA synthesis

Leaf samples were collected from tomato plants damaged by VEGI caterpillars, VEGA caterpillars, mechanically injured (MI) plants, mechanical injured plants treated with OS from VEGI caterpillars (MI + OSVEGI), mechanical injured plants treated with OS from VEGA caterpillars (MI + OSVEGA), and undamaged (control plants), after 12 h of caterpillars feeding. Leaf samples were immediately frozen in liquid nitrogen and kept at -80°C. Frozen samples were ground to a fine powder in liquid nitrogen with a pestle and mortar. Total RNA was extracted from 100 mg of each leaf sample using Spectrum™ plant total RNA kit (Sigma Aldrich, St. Louis, MO, USA), according to the manufacturer’s instructions. RNA concentration and purity were determined using a NanoDrop™ Spectrophotometer ND-2000 (Thermo Scientific, Wilmington, DE, USA), and the integrity of RNA was also assessed by 1% agarose gel electrophoresis and ethidium bromide staining. The absence of contaminant DNA in the RNA samples was verified by PCR using specific primers of a known gene and gel electrophoresis analysis. No fragments of genomic DNA were identified in all samples tested in this work. First strand cDNA was synthesized from 200 ng RNA using a Goscrpit™ Reverse Transcription System Kit (Promega, Madison, WI, USA) according to the manufacturer’s instructions.

### Real-time PCR

The transcript levels of genes that are involved in tomato defense signaling pathway, such as lipoxygenase (*LOX2*), allene oxide synthase (*AOS*), and four terpene synthase (*TPS*) genes, were measured by quantitative RT-PCR (see list of primers used in Table 4). Quantitative real-time PCR (qrtPCR) was carried out on an ABI 7500 Real Time PCR System (Life Technologies, Carlsbad, CA, USA) with a 96 well rotor. The amplification reactions were performed with 25 μl of mixture consisting of 12.5 μl of PerfeCTA® SYBR® Green Fastmix® LOW ROX qPCR Master Mix (Quanta Biosciences, Inc, Gaithersburg, MD, USA), 0.5 μl of cDNA and 100 nM primers (Integrated DNA Technologies, Coralville, IA, USA). Relative RNA levels were calibrated and normalized with the level of two housekeeping genes: *Actin* and *18S* ribosomal mRNA. PCR conditions were determined by comparing threshold values in a dilution series of the RT product, followed by non-template control for each primer pair. Relative expression levels of genes were calculated by using the Pfaffl method [67]. A suitable melt curve analysis was also performed. The data were analyzed by using one-way ANOVA followed by the Tukey-Kramer HSD multiple comparison test at a significance level of *P* < 0.05.

**Table 4:** Primers used for RT-qPCR

**Genes**	**Direction**	**Primer sequences (5′-3′)**	**GenBank (AN)**	**References**
*AOS*	Forward	GGGTGAAATCCTATTCGGGT	AF230371	[68]
	Reverse	CGCACTGTTTATTCCCCACT		
*LOX2*	Forward	TGCAACACGCACCATTTATT	U37840	[50]
	Reverse	GTGACAACACGTTTGGATCG		
*TPS5*	Forward	CTATTTCCACCACAAGGCGT	AY840091	[37]
	Reverse	TTCATCATGTGATCCCTCCA		
*TPS12*	Forward	GCCCAATGGTTAAACAATGATAATC	JN412092	[37]
	Reverse	ATATAACGTGTTTATCACGCGTGTG		
*TPS25*	Forward	GTGGGTCAACTTCTGTAAAGCTTTAC	JN412085	[37]
	Reverse	TGATTAACAATTTTTTTCTGTGATGTT		
*TPS7*	Forward	CAAGGAGTATGTTAATGTCAGG	JN412082	[37]
	Reverse	GCTTCATATAAGTTCAATATTCC		

Allene oxide synthase (*AOS*), Lipoxygenase (*LOX2*), Tomato monoterpene synthase 5 *(TPS5),* Tomato terpene synthase 9 *(TPS9),* and Tomato terpene synthase 12 *(TPS12).* Accession number = *AN*.

## Abbreviations

VEG: Ventral eversible gland; VOCs: Volatile organic compounds; ROS: Reactive oxygen species; MI: Mechanical injury; VEGI: VEG Intact; VEGA: VEG ablated; OS: Oral secretion; AOS: Allene oxide synthase; LOX: Lipoxygenase; TPS: Terpene synthases; MI + OSVEGI: Mechanically injured plants treated with OS from VEGI caterpillars; MI + OSVEGA: Mechanically injured plants treated with OS from VEGA caterpillars.

## Competing interests

The authors declare that they have no competing interests.

## Authors’ contributions

HF and SZ designed the study. SZ, JP and JD performed the research. SZ analyzed the data. HF and SZ wrote the paper. All authors read and approved the final manuscript.
